# Outcomes of Acute Type A Aortic Dissection in Octogenarians: Tokyo Acute Aortic Super-Network Registry

**DOI:** 10.1093/icvts/ivag195

**Published:** 2026-07-14

**Authors:** Manabu Yamasaki, Hideaki Yoshino, Takashi Kunihara, Kojiro Morita, Koichi Akutsu, Tomoki Shimokawa, Hitoshi Ogino, Mitsuhiro Kawata, Toshiyuki Takahashi, Michio Usui, Takeshiro Fujii, Shun Kohsaka, Takeshi Yamamoto, Morimasa Takayama

**Affiliations:** Department of Cardiovascular Surgery, Juntendo University, 2-2-1 Hongo, Bunkyoku, Tokyo, 113-8421, Japan; Department of Cardiovsascular Surgery, Itabashi Chuo Medical Hospital, 2-12-17 Azusawa, Itabashiku, Tokyo, 174-0051, Japan; Tokyo CCU Network Scientific Committee, 2-23-1 Yoyogi, Shibuyaku, Tokyo151-0053, Japan; Tokyo CCU Network Scientific Committee, 2-23-1 Yoyogi, Shibuyaku, Tokyo151-0053, Japan; Department of Nursing Administration and Advanced Clinical Nursing, Tokyo University, 7-3-1 Hongo, Bunkyoku, Tokyo, 113-0033, Japan; Tokyo CCU Network Scientific Committee, 2-23-1 Yoyogi, Shibuyaku, Tokyo151-0053, Japan; Tokyo CCU Network Scientific Committee, 2-23-1 Yoyogi, Shibuyaku, Tokyo151-0053, Japan; Tokyo CCU Network Scientific Committee, 2-23-1 Yoyogi, Shibuyaku, Tokyo151-0053, Japan; Tokyo CCU Network Scientific Committee, 2-23-1 Yoyogi, Shibuyaku, Tokyo151-0053, Japan; Tokyo CCU Network Scientific Committee, 2-23-1 Yoyogi, Shibuyaku, Tokyo151-0053, Japan; Tokyo CCU Network Scientific Committee, 2-23-1 Yoyogi, Shibuyaku, Tokyo151-0053, Japan; Tokyo CCU Network Scientific Committee, 2-23-1 Yoyogi, Shibuyaku, Tokyo151-0053, Japan; Tokyo CCU Network Scientific Committee, 2-23-1 Yoyogi, Shibuyaku, Tokyo151-0053, Japan; Tokyo CCU Network Scientific Committee, 2-23-1 Yoyogi, Shibuyaku, Tokyo151-0053, Japan; Tokyo CCU Network Scientific Committee, 2-23-1 Yoyogi, Shibuyaku, Tokyo151-0053, Japan

**Keywords:** acute aortic dissection, octogenarians, network

## Abstract

**Objectives:**

The increasing prevalence of acute Stanford type A aortic dissection (ATAAD) among octogenarians necessitates robust, large-scale data to inform surgical decision-making and risk stratification. Thus, we investigated the multicentre Tokyo Acute Aortic Super-Network database to demonstrate in-hospital mortality trends in this vulnerable patient population.

**Methods:**

We analysed patients with ATAAD transported to the network’s participating hospitals between January 2015 and December 2022. The study population was stratified into 2 age-based groups: octogenarians (≥80 years) and non-octogenarians (<80 years). Multivariate logistic regression analysis identified independent predictors of in-hospital mortality specifically within the octogenarian group.

**Results:**

Among 5361 patients, 1467 (27.4%) were octogenarians. Overall mortality was significantly higher in the octogenarians compared to non-octogenarians (34.5% vs 15.9%; *P* < .001), whereas operative mortality for those undergoing surgery remained acceptable (10.5% vs 8.1%; *P* = .035). Multivariable analysis revealed that open false lumen (odds ratio [OR], 3.77; 95% confidence interval [CI], 2.51-5.67; *P* < .001), shock at arrival (OR, 2.62; 95% CI, 1.68-4.08; *P* < .001), out-of-hospital cardiopulmonary arrest (OR, 18.99; 95% CI, 7.72-46.72; *P* < .001), in-hospital cardiopulmonary arrest (OR, 17.39; 95% CI, 6.21-48.67; *P* < .001), and cerebral malperfusion (OR, 2.17; 95% CI, 1.16-4.06; *P* = .016) were related to higher mortality in octogenarian patients. Conversely, surgical intervention (OR, 0.15; 95% CI, 0.10-0.22; *P* < .001) and higher serum albumin levels (OR, 0.65; 95% CI, 0.42-1.00; *P* = .049) were associated with lower risk of mortality.

**Conclusions:**

Although octogenarians with ATAAD had higher overall mortality, operative mortality among selected patients undergoing surgery remained acceptable, suggesting that advanced age alone should not preclude surgical intervention.

## Graphical abstract

**Figure:**
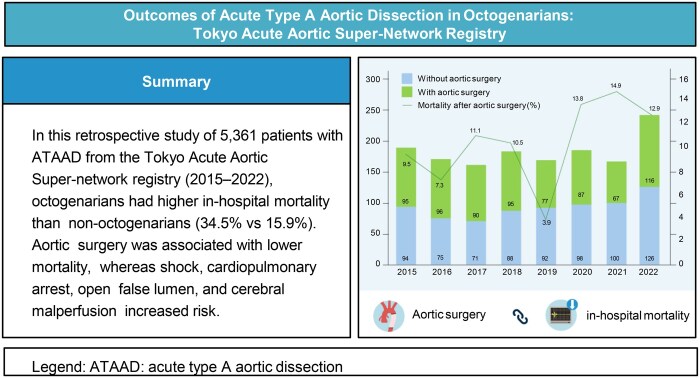

## INTRODUCTION

Currently, life expectancy in Japan exceeds 80 years, and approximately 10% of the population is aged ≥80 years.^1^ Many octogenarians present with acute aortic dissection (AAD). We previously reported that octogenarian status and medical therapy were associated with early mortality in acute Stanford type A aortic dissection (ATAAD).^2^ Data from the Tokyo Acute Aortic Super-Network (TAAS) registry similarly demonstrated early mortality after surgery for ATAAD,^3^ consistent with findings from high-volume Japanese centres.^4^

The TAAS registry, established in 2010, provides a coordinated transfer system for patients with suspected or confirmed acute aortic syndrome (AAS), including ATAAD, and reduces prehospital delays through ambulance coordination under the Tokyo Fire Department.^5^ Currently, 42 hospitals participate in the network.

A lower proportion of octogenarians undergo surgery compared with younger patients, likely reflecting differences in comorbidities, physiological reserve, and treatment selection. Therefore, we investigated the outcomes and predictors of in-hospital mortality among octogenarians with ATAAD transported to TAAS hospitals.

## PATIENTS AND METHODS

### Ethical statement

This study complied with the Declaration of Helsinki and Japanese government guidelines. The Tokyo Acute Aortic Super-Network registry is managed in accordance with the WMA Declaration of Taipei. The database is continuously maintained under the oversight of the institutional review boards of participating hospitals, including the Itabashi Chuo Medical Centre, which approved this study (Approval No. 250624c). Individual informed consent was waived because only de-identified data were used.

### Study database and definitions

Patients diagnosed with ATAAD and transported via the TAAS between 2015 and 2022 were included. Data were collected from emergency departments, cardiovascular surgery units, and critical care units using standardized forms. TAAS comprises 14 high-volume and 28 support centres in Tokyo.

### Primary and secondary end-points

The primary end-point was in-hospital mortality in octogenarians compared with non-octogenarians. Secondary analyses evaluated predictors of mortality in octogenarians and annual trends in surgical treatment and mortality from 2015 to 2022. Outcomes of octogenarians managed without surgery were also assessed.

### Definition of co-variables

Classic dissection was defined as a non-thrombosed false lumen, and intramural haematoma (IMH) as a thrombosed lesion. Out-of-hospital cardiopulmonary arrest (OHCPA) and in-hospital cardiopulmonary arrest (IHCPA) were diagnosed when CPA was confirmed by ambulance personnel or hospital staff. Return of spontaneous circulation (ROSC) was defined as a palpable pulse or measurable blood pressure. Coronary, cerebral, and visceral malperfusion were diagnosed based on electrocardiography or biomarker evidence, neurological deficits with computed tomography (CT), and CT findings of ischaemia with supportive data. Preoperative extracorporeal membrane oxygenation (ECMO) was used to manage cardiogenic shock. Time from onset to admission was defined as the interval from symptom onset to arrival at the first hospital. Consciousness was assessed using the Japan Coma Scale (JCS), and neurological deficit was defined as any disturbance other than JCS 0.

### Statistical analyses

Comparisons between groups used chi-square or Fisher’s exact tests for categorical variables and Student’s *t*-test or Mann-Whitney *U*-test for continuous variables. Multivariable logistic regression identified predictors of in-hospital mortality in octogenarians, reported as odds ratios (ORs) and 95% confidence intervals (CIs). Missing data were handled using multiple imputation with chained equations, generating 20 datasets combined using Rubin’s rules. Age was also modelled as a continuous variable using restricted cubic splines (RCS) with 4 knots. Poisson regression was used to assess annual case numbers, and Cochran-Armitage tests evaluated trends in surgery and postoperative mortality. Penn classification^6^ was applied to surgical patients based on their preoperative status to compare outcomes between octogenarians and non-octogenarians according to physiological severity. Statistical significance was set at *P* < .05. Analyses were performed using SPSS version 24 (IBM Corp., Chicago, IL, United States).

## RESULTS

### Baseline characteristics of patients with ATAAD, comparison between octogenarians and non-octogenarians

**Figure 1.**
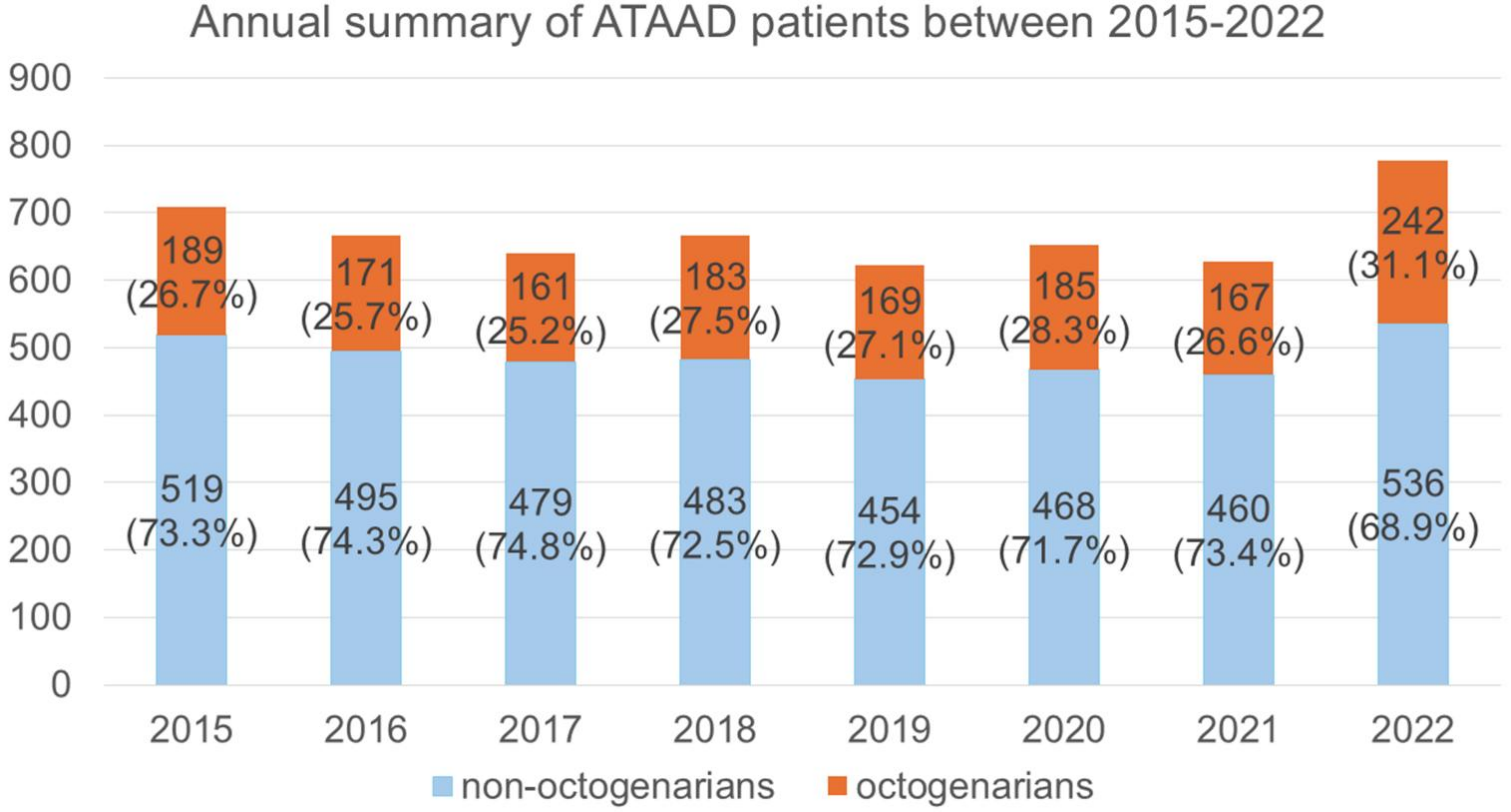
Summary of Patients With ATAAD Between 2015 and 2022

Among 5361 ATAAD cases, 1467 (27.4%) involved octogenarians (**Table 1**). Their proportion remained around 30% in 2018-2022. A significant annual increase was found in octogenarian cases (*β* = 0.0266, *P* = .019), corresponding to ∼2.7% per year (**Figure 1**).

**Table 1.** Baseline Characteristics of Patients With ATAAD

	Total	Octogenarians	Non-octogenarians	*P*
	(*n* = 5361)	(*n* = 1467)	(*n* = 3894)	
Age (years), mean (SD)	69.2 (14.1)	85.5 (4.29)	63.0 (11.4)	<.001
Male, *n* (%)	2838 (52.9)	415 (28.3)	2423 (62.2)	<.001
Hypertension, *n* (%)	3246 (60.5)	892 (60.8)	2534 (60.2)	.814
Hyperlipidaemia, *n* (%)	863 (16.1)	250 (17.0)	613 (15.7)	.248
Diabetes mellitus, *n* (%)	345 (6.4)	110 (7.5)	235 (6.0)	.052
Previous coronary disease, *n* (%)	217 (4.0)	87 (5.9)	130 (3.3)	<.001
Previous stroke, *n* (%)	405 (7.6)	156 (10.6)	249 (6.4)	<.001
Previous heart failure, *n* (%)	98 (1.8)	52 (3.5)	46 (1.2)	<.001
DeBakey classification type I, *n* (%)	4086 (76.2)	1003 (68.4)	3083 (79.2)	<.001
Classic type aortic dissection, *n* (%)	3209 (84.4)	752 (51.3)	2457 (63.1)	<.001
Shock at hospital arrival, *n* (%)	1296 (24.2)	461 (31.5)	832 (21.5)	<.001
Out-of-hospital CPA (OHCPA), *n* (%)	471 (8.8)	196 (13.4)	275 (7.1)	<.001
In-hospital CPA (IHCPA), *n* (%)	209 (3.9)	71 (4.8)	138 (3.5)	.029
Use of ECMO, *n* (%)	354 (6.7)	54 (3.7)	300 (7.8)	<.001
Coronary malperfusion, *n* (%)	458 (8.5)	125 (8.5)	333 (8.6)	.971
Cerebral malperfusion, n (%)	327 (6.1)	84 (5.7)	243 (6.2)	.483
Visceral malperfusion, *n* (%)	59 (1.1)	10 (0.7)	49 (1.3)	.074
Aortic surgery, *n* (%)	3636 (67.8)	723 (49.3)	2913 (74.8)	<.001
Onset to hospital (h), median (IQR)	2.20 (0.90-4.00)	2.00 (0.83-4.28)	2.25 (0.90-3.84)	.005
Mortality, *n* (%)	1124 (21.0)	506 (34.5)	618 (15.9)	<.001
Mortality after aortic surgery, *n* (%)	311 (8.6)	76 (10.5)	236 (8.1)	.035

Abbreviations: CPA, cardiopulmonary arrest; classic-type aortic dissection, false lumen not thrombosed in the dissected thoracic aorta; ECMO, extracorporeal membrane oxygenation.

Octogenarians had lower rates of classic dissection (51.3% vs 63.1%; *P* < .001) and DeBakey type I (68.4% vs 79.2%; *P* < .001) but more previous coronary disease, heart failure, and prior stroke cases (all *P* < .001) and more frequently presented with shock (31.5% vs 21.5%), OHCPA (13.4% vs 7.1%), and IHCPA (4.8% vs 3.5%) than non-octogenarians. Surgery and ECMO use were lower in octogenarians (49.3% vs 74.8% and 3.7% vs 7.8%). In-hospital mortality was higher (34.5% vs 15.9%; *P* < .001), including after surgery (10.5% vs 8.1%; *P* = .035). Malperfusion rates were similar between the groups.

### Comparison between deceased and surviving octogenarians

#### Univariable analysis

**Figure 2.**
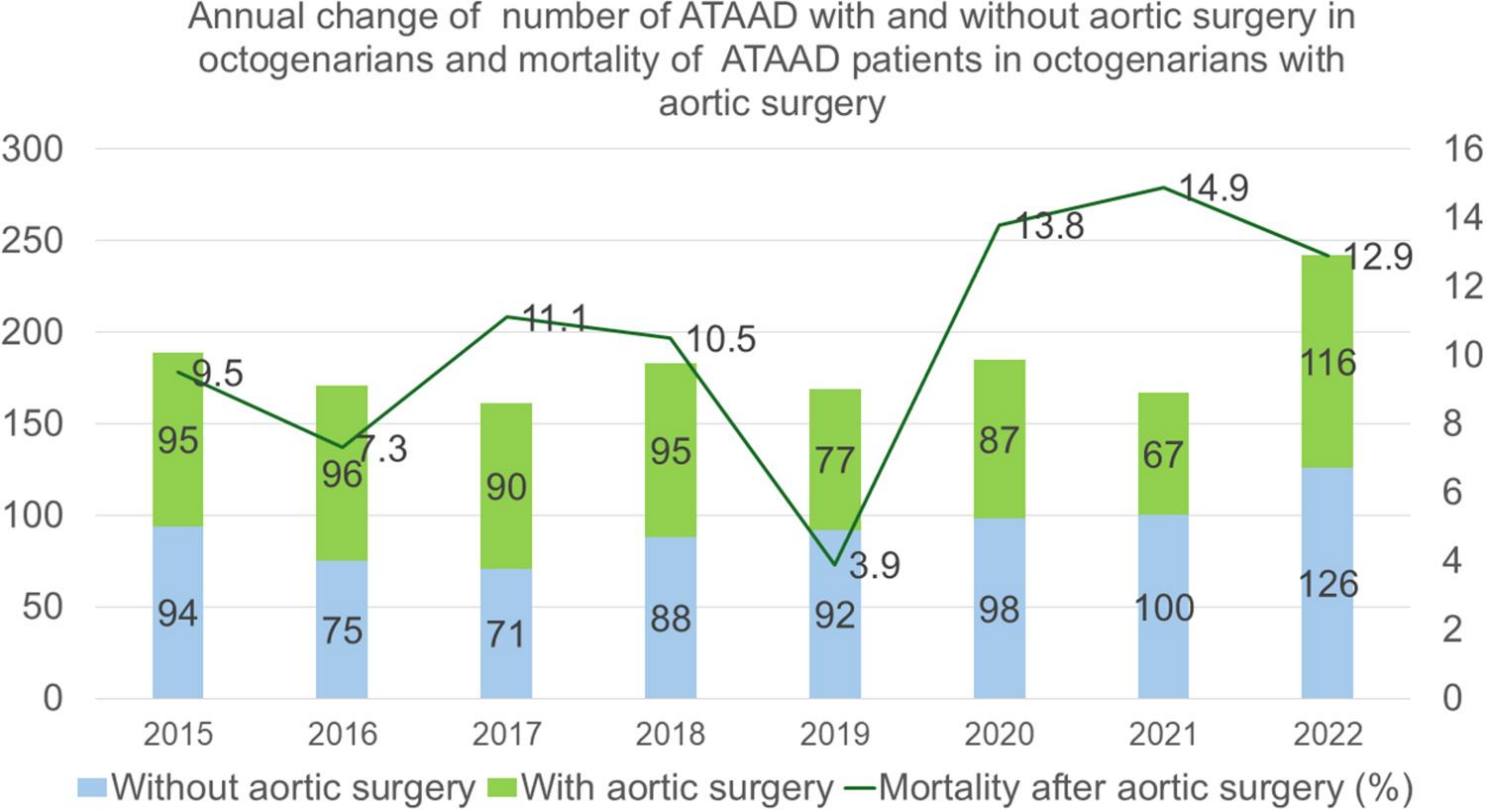
Annual Change in the Number of Patients With ATAAD, With and Without Aortic Surgery in Octogenarians, and Mortality of Octogenarian Patients With ATAAD Who Underwent Aortic Surgery

Among octogenarians (**Table 2**), non-survivors had higher classic dissection, DeBakey type I, coronary disease, and heart failure rates and lower hypertension and hyperlipidaemia rates. Shock (63.4% vs 14.6%), OHCPA (37.4% vs 0.7%), and IHCPA (13.0% vs 0.5%) were markedly more common in non-survivors (all *P* < .001). Surgery was performed more often in survivors (67.3% vs 15.0%). Coronary and cerebral malperfusion were more frequent in non-survivors. Between 2015 and 2022, surgical rates increased over time (Z = 2.50, *P* = .012), whereas operative mortality did not (*P* = .13) (**Figure 2**).

**Table 2.** Univariable and Multivariable Analysis of Predictors for In-Hospital Mortality of Octogenarian Patients With ATAAD

	Total (*n* = 1467)	Survived (*n* = 961)	Deceased (*n* = 506)	*P-*value	Adjusted odds ratio	95% CI	*P-*value
Age (years), mean (SD)	85.5 (4.29)	84.8 (3.87)	86.8 (4.74)	<.001	1.12	1.08-1.17	<.001
Male, *n* (%)	415 (28.3)	263 (27.4)	152 (30.0)	.28	1.19	0.81-1.74	.383
Hypertension, *n* (%)	892 (60.8)	632 (65.8)	260 (51.4)	<.001	0.79	0.56-1.13	.195
Hyperlipidaemia, *n* (%)	250 (17.0)	193 (20.1)	57 (11.3)	<.001	1.04	0.67-1.64	.850
Diabetes mellitus, *n* (%)	110 (7.5)	70 (7.3)	40 (7.9)	.67	1.14	0.63-2.06	.42
Previous coronary disease, *n* (%)	87 (5.9)	48 (5.0)	39 (7.7)	.037	1.41	0.74-2.69	.302
Previous stroke, *n* (%)	156 (10.6)	101 (10.5)	55 (10.9)	.83	1.57	0.96-2.58	.072
Previous heart failure, *n* (%)	52 (3.5)	27 (2.8)	25 (4.9)	.036	1.76	0.79-3.92	.163
DeBakey classification Type I, *n* (%)	1003 (68.4)	640 (66.6)	363 (71.7)	<.001	0.93	0.64-1.33	.680
Classic type aortic dissection, *n* (%)	752 (51.3)	454 (47.2)	298 (58.9)	<.001	3.77	2.51-5.67	<.001
Shock at hospital arrival, *n* (%)	461 (31.5)	140 (14.6)	321 (63.4)	<.001	2.62	1.68-4.08	<.001
Out-of-hospital CPA (OHCPA), *n* (%)	196 (13.4)	7 (0.7)	189 (37.4)	<.001	18.99	7.72-46.72	<.001
In-hospital CPA (IHCPA), *n* (%)	71 (4.8)	5 (0.5)	66 (13.0)	<.001	17.39	6.21-48.67	<.001
Use of ECMO, *n* (%)	54 (3.7)	37 (3.9)	17 (3.4)	.64	1.19	0.43-3.27	.735
Coronary malperfusion, *n* (%)	125 (8.5)	47 (4.9)	78 (15.4)	<.001	1.70	0.90-3.19	.101
Cerebral malperfusion, *n* (%)	84 (5.7)	43 (4.5)	41 (8.1)	.004	2.17	1.16-4.06	.016
Visceral malperfusion, *n* (%)	10 (0.7)	5 (0.5)	5 (1.0)	.30	0.93	0.09-9.57	.950
Aortic surgery, *n* (%)	723 (49.3)	647 (67.3)	76 (15.0)	<.001	0.15	0.10-0.22	<.001
Onset to hospital (h), median (IQR)	2.00 (0.83-4.28)	2.90(1.10-5.30)	1.00(0.70-2.13)	<.001	0.96	0.92-1.01	.092
Hb (g/dL) (SD) on admission	11.3 (1.84)	11.4 (1.82)	11.1 (1.86)	.004	1.02	0.90-1.14	.782
CK (IU/L) (SD) on admission	151.0 (437.1)	117.7 (435.7)	215.3 (433.0)	<.001	1.00	1.00-1.00	.102
CRP (mg/dL) (SD) on admission	2.58 (5.31)	2.81 (5.56)	2.13 (4.76)	.024	0.99	0.96-1.03	.714
D-dimer (μg/mL) (SD) on admission	66.3 (292.2)	30.3 (65.9)	137.9 (489.3)	<.001	1.00	1.00-1.00	.201
Albumin (g/dL) (SD) on admission	3.36 (0.54)	4.47 (5.23)	2.37 (3.68)	<.001	0.65	0.42-1.00	.049

Abbreviations: CK, creatine kinase; CPA, cardiopulmonary arrest; CRP, C-reactive protein; ECMO, extracorporeal membrane oxygenation; Hb, haemoglobin.

#### Multivariable analysis of in-hospital mortality predictors in octogenarians with ATAAD

Open false lumen (OR, 3.77; 95% CI, 2.51-5.67; *P* < .001), shock at arrival (OR, 2.62; 95% CI, 1.68-4.08; *P* < .001), OHCPA (OR, 18.99; 95% CI, 7.72-46.72; *P* < .001), IHCPA (OR, 17.39; 95% CI, 6.21-48.67; *P* < .001), and cerebral malperfusion (OR, 2.17; 95% CI, 1.16-4.06; *P* = .016) were associated with higher mortality in octogenarian patients with ATAAD; however, higher albumin (OR, 0.65; 95% CI, 0.42-1.00; *P* = .049) and aortic surgery (OR, 0.15; 95% CI, 0.10-0.22; *P* < .001) were associated with lower mortality (**Table 2**).

#### Without aortic surgery in octogenarian patients with ATAAD

**Figure 3.**
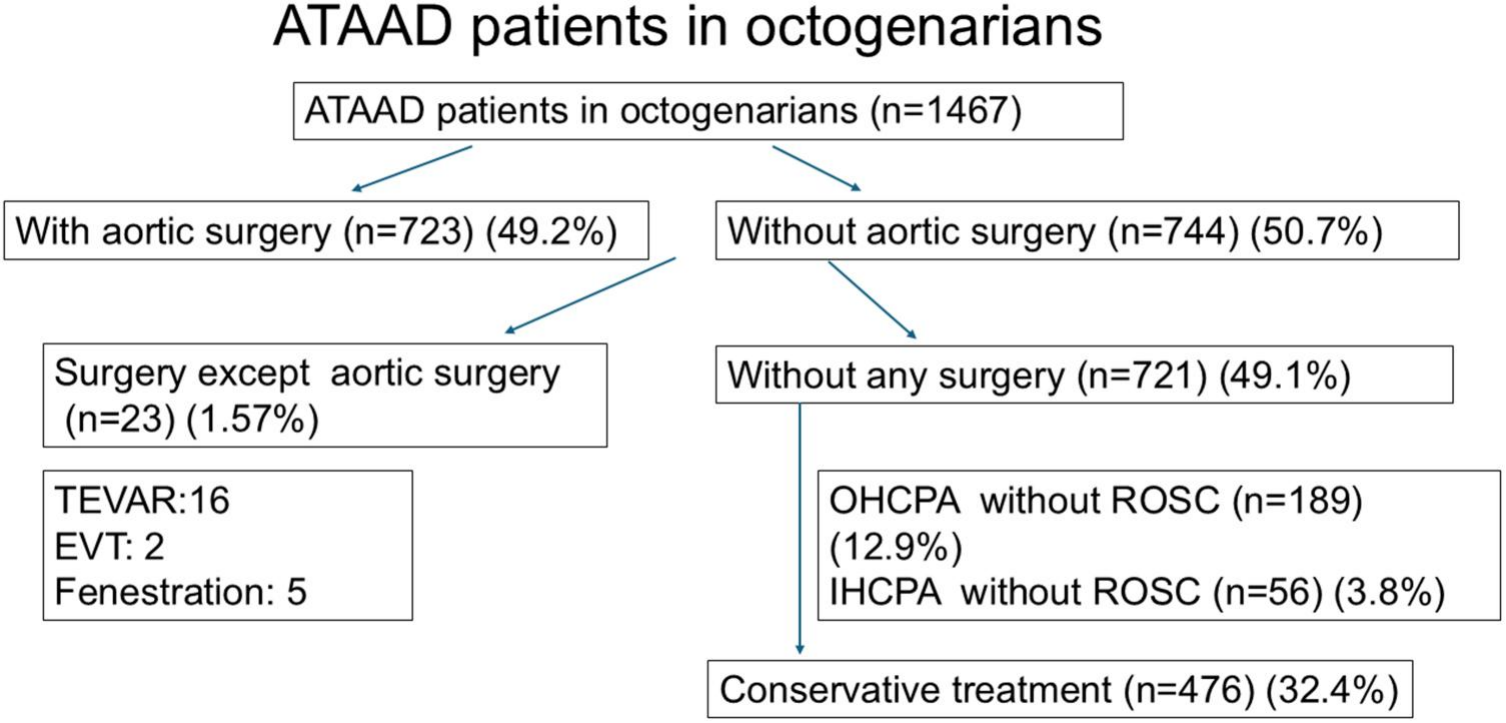
Octogenarian Patients With ATAAD

After excluding 723 aortic surgeries (49.2%), 23 endovascular repairs (stent graft: 16, endovascular treatment: 2, fenestration: 5), and 189 OHCPA and 56 IHCPA cases without ROSC, 476 octogenarians (32.4%) underwent conservative treatment (**Figure 3**). Among these patients (**Table 3**), age (OR 1.16; 95% CI 1.02-1.31; *P* = .019), shock on arrival (OR 4.10; 95% CI 1.45-11.62; *P* = .008), and neurological deficit (OR 3.56; 95% CI 1.44-8.76; *P* = .006) independently predicted higher mortality, whereas IMH was associated with lower mortality (OR 0.27; 95% CI 0.11-0.65; *P* = .004).

**Table 3.** Octogenarian Patients With Conservative Treatment for *ATAAD*

	**All (*n*** = **476)**	Survived (*n* = 297)	Deceased (*n* = 179)	*P*-value	Adjusted odds ratio	95% CI	*P*-value
Age (SD)	87.2 (4.56)	86.3 (4.41)	88.7 (4.46)	<.001	1.16	1.02-1.31	.019
Over 85, *n* (%)	331 (69.8)	186 (62.8)	145 (81.5)	<.001	2.92	0.75-11.41	.124
Male, *n* (%)	131 (27.5)	90 (30.3)	41(22.9)	.080	1.51	0.60-3.80	.376
IMH, *n* (%) (*n* = 777)	183 (49.5)	149 (63.4)	34 (25.2)	<.001	0.27	0.11-0.65	.004
Previous coronary disease, *n* (%)	33 (6.9)	20 (6.7)	13 (7.3)	.83	0.92	0.22-3.92	.915
Previous heart failure, *n* (%)	23 (4.8)	11 (3.7)	12 (6.7)	.14	1.45	0.25-8.58	.084
Previous CVA, *n* (%)	56 (11.8)	32 (10.8)	24 (13.4)	.39	3.63	1.00-13.16	.05
Previous HT, *n* (%)	301 (63.2)	196 (66.0)	105 (58.7)	.11	0.97	0.41-2.26	.93
Previous HL, *n* (%)	83 (17.4)	54 (18.2)	29 (16.2)	.58	1.83	0.65-5.20	.26
Shock on arrival, *n* (%)	96 (20.2)	33 (11.1)	63 (35.2)	<.001	4.10	1.45-11.62	.008
Neurological deficit, *n* (%)	248 (54.4)	118 (42.0)	130 (74.3)	<.001	3.56	1.44-8.76	.006
Hb (g/dL) (SD)	11.6 (1.8)	11.7 (1.8)	11.3 (1.8)	.007	0.99	0.79-1.23	.99
CK (IU/L) (SD)	165.6 (679.6)	145.6 (772.0)	198.2 (492.8)	.374	1.003	1.00-1.01	.22
CRP (mg/dL) (SD)	3.23 (6.28)	3.41(6.50)	2.95 (5.89)	.434	0.96	0.89-1.04	.32
D dimer (μg/mL) (SD)	36.4 (87.9)	25.8 (67.4)	55.6 (114.1)	.006	1.003	0.31-1.72	.23
Albumin (g/dL) (SD)	3.44 (0.52)	3.49 (0.51)	3.34 (0.51)	.012	0.734	0.31-1.72	.48

Abbreviations: CK, creatine kinase; CRP, C-reactive protein; CVA, cerebral vascular accident; Hb, haemoglobin; IMH, intramural haematoma; HT, hypertension; HL, hyperlipidemia.

Restricted cubic splines analysis showed that mortality remained stable at younger ages but rose sharply beyond ∼80 years, with no plateau or secondary inflection point (**Figure S1**).

#### Comparison between non-octogenarians and octogenarians according to Penn classification

**Figure 4.**
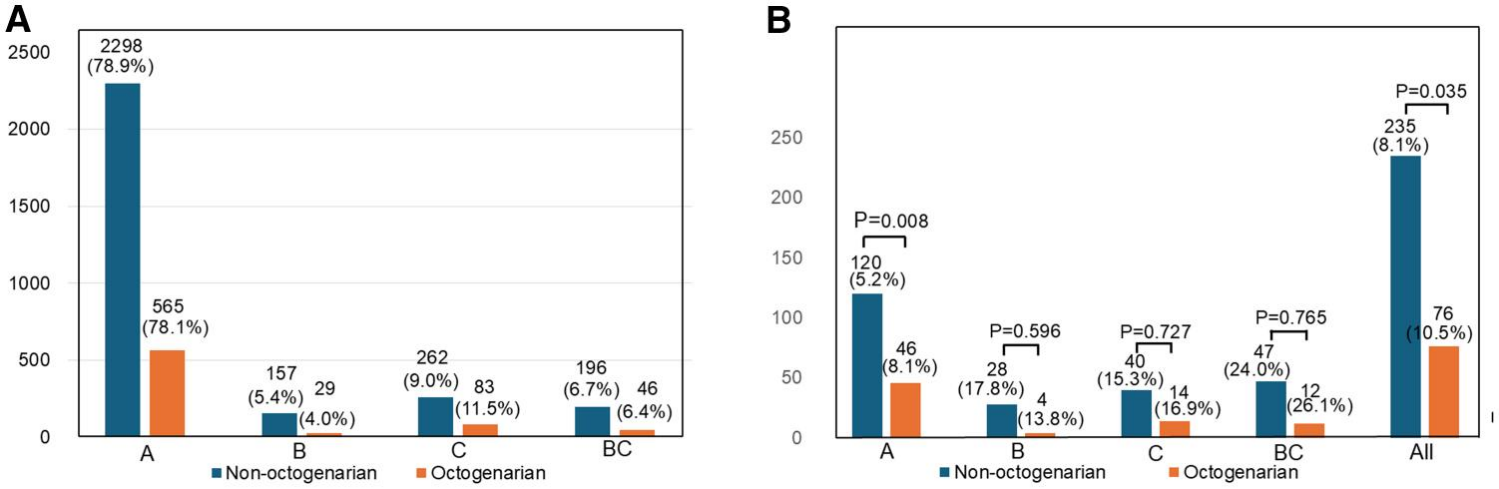
Distribution of PENN classes (A) and in-hospital mortality (B) among non-octogenarian and octogenarian patients who underwent aortic surgery. Panel A shows the distribution of PENN classes (A, B, C, BC). A: Haemodynamically stable without signs or symptoms of ischaemia; B: haemodynamically stable with branch-vessel malperfusion; C: global malperfusion due to circulatory collapse; BC: global malperfusion with evidence of local ischaemia. Panel B shows in-hospital mortality stratified by the same PENN classes

The distribution of non-octogenarian and octogenarian patients who underwent aortic surgery according to Penn classification is shown in **Figure 4A** (A: 78.9% vs 78.1%, B: 5.4% vs 4.0%, C: 9.0% vs 11.5%, and BC: 6.7% vs 6.4%). In-hospital mortality after aortic surgery differed significantly between non-octogenarian and octogenarian patients in Penn class A (5.2% vs 8.1%; *P* = .0088) but not in Penn classes B, C, and BC (**Figure 4B**).

## DISCUSSION

### Comparison between octogenarians and non-octogenarians with ATAAD

The higher ATAAD incidence^7^ and Japan’s long-life expectancy suggest a growing number of octogenarian patients. Reported operative mortality for ATAAD was lower in Japan (9.5%-11.0%)^8–10^ than in international registries (16.9%-18.4%).^11–13^ Our registry showed a lower rate (8.5%). However, because detailed procedural variables and standardized risk adjustment measures were unavailable, these observations should be interpreted cautiously. Although transfer efficiency or population characteristics, including smaller body size,^2^ may contribute, these explanations remain speculative, and direct comparisons with Western registries are limited.

Regardless of surgical intervention, all patients with ATAADs transported to TAAS network hospitals were included. Octogenarians comprised approximately 30% of cases from 2015 to 2022, with no marked upward trend. In contrast, the increasing proportion of octogenarians undergoing surgery, together with the absence of a corresponding rise in operative mortality, may indicate that a larger number of octogenarian patients retain sufficient physiological reserve to tolerate major aortic surgery. This trend may reflect advances in surgical and perioperative management that have enabled safe operative treatment in an expanding older adult population.

Overall male proportion was 52.9%, with 62.2% among non-octogenarians and only 28.3% among octogenarians—likely reflecting female longevity. Compared with younger patients, octogenarians had more comorbidities (coronary and cerebrovascular disease), contributing to higher shock rates.

The lower prevalence of classic type and DeBakey type I dissections in octogenarians may reflect reduced extension to supra-aortic and abdominal vessels with age.^14^ This observation aligns with recent findings showing octogenarians often present with DeBakey type II dissection and IMH compared with younger patients.^15^ ECMO was used more frequently in non-octogenarians, suggesting aggressive resuscitation in younger patients.^16^

Acute Stanford type A aortic dissection mortality rate was 21% overall, with 34.5% in octogenarians and 15.9% in non-octogenarians. Among surgically treated cases, mortality was 10.5% vs 8.0%, respectively—this modest but significant 2.5% difference may reflect treatment selection and differences in surgical strategy. Many octogenarians have primary entry into the ascending aorta, allowing for less invasive procedures.^17^

Octogenarians had shorter times from symptom onset to hospital admission despite presenting more frequently with shock, OHCPA, and IHCPA. This likely reflects delayed ATAAD recognition, as symptoms in older adults are often non-specific. Comparing the outcomes of octogenarians and non-octogenarians who underwent aortic surgery using Penn classification (**Figure 4A and B**), it was observed that the outcomes of non-octogenarians were better in only Penn class A, which was the most stable state. This suggests that even in haemodynamically stable presentations, older adult patients may harbour greater physiological vulnerability and reduced organ reserve.

The present analysis of Penn classification provides important insight into age-dependent differences in surgical outcomes. Although octogenarians and non-octogenarians showed similar Penn class distributions, mortality differences appeared only in Penn class A, the haemodynamically stable group without malperfusion. This suggests age has greater impact when physiological derangement is minimal. Once malperfusion or collapse develops, mortality appears driven mainly by ischaemic severity rather than age, resulting in similar outcomes across age groups. These findings underscore the importance of early recognition and timely referral before the onset of malperfusion, as age-related vulnerability may be most pronounced in clinically stable presentations. Therefore, Penn class A octogenarians may benefit from proactive operative management, whereas patients presenting with advanced malperfusion require individualized assessment that balances ischaemic severity and physiological reserve.

The RCS analysis further supports these observations. The risk of in-hospital mortality remained relatively stable across younger age groups but increased sharply beyond approximately 80 years of age. Together, these results suggest that the decline in physiological reserve associated with advanced age becomes most evident in patients who present without overt ischaemia or circulatory collapse. Conversely, in the presence of severe malperfusion, the ischaemic burden appears to outweigh the influence of chronological age as the principal determinant of mortality.

### ATAAD in octogenarian patients

The proportion of octogenarians undergoing surgery increased over time without a corresponding increase in operative mortality, suggesting that surgical treatment is being offered to a broader older population while maintaining acceptable outcomes.

International Registry of Acute Aortic Dissection (IRAD) data suggested comparable surgical risks between octogenarians and younger cohorts, although mortality of octogenarians remained high (25.1%).^18^ Conversely, the in-hospital mortality rates of aortic surgery for ATAAD in octogenarians were higher than in non-octogenarians in Japanese large-scale registries such as Japan Cardiovascular Surgery Database, Japanese Registry of Acute Aortic Dissection, and our registry, which reported that the in-hospital mortality rate of aortic surgery in octogenarians was 10%-12%.^4^^,^^10^ The superior outcomes observed in Japan may be partly attributable to differences in medical systems, including interhospital patient transfer protocols, compared to those in Western countries.

Surgical decision-making in octogenarians is influenced by age, comorbidities, frailty, and cognitive function. Japanese surgeons often favour proximal repair because dissection is frequently localized. Frozen elephant trunk procedures are increasingly used to reduce long-term aortic events.^19^ This may have contributed to the small difference in in-hospital mortality between octogenarians and non-octogenarians.

Multivariable analysis identified age, classic-type dissection, cerebral malperfusion, OHCPA, IHCPA, and shock at presentation as independent predictors of higher in-hospital mortality. In contrast, higher serum albumin levels and surgical intervention were independently associated with lower mortality. Patients with IMH had lower mortality than those with classic-type dissection, including among patients managed conservatively.^20^^,^^21^

International Registry of Acute Aortic Dissection and German Registry for Acute Aortic Dissection Type A (GERAADA) were used to confirm coronary and cerebral malperfusion as key risks.^22^^,^^23^ Our data showed that cerebral malperfusion was significant; however, coronary malperfusion was not. This may be due to prehospital CPA,^3^ as it is well known that older patients are vulnerable to coronary ischaemia. Higher albumin levels were associated with lower mortality and may reflect better nutritional and physiological reserve.^24^

These findings support an individualized treatment approach for octogenarians. Although advanced age was associated with higher mortality, operative mortality remained acceptable in selected surgical candidates. Shock, cardiopulmonary arrest, cerebral malperfusion, and low albumin levels may help identify patients at increased risk.

Our findings should be interpreted in the context of the landmark study by Kreibich et al,^25^ which evaluated 1187 patients with ATAAD using the Penn classification and demonstrated that advancing age substantially increased mortality risk across all Penn classes. In their North American cohort, predicted mortality increased progressively with age, reaching approximately 25% in Penn classes B and C and exceeding 50% in Penn class BC among older patients. In contrast, the TAAS registry demonstrated similar distributions of Penn classes between octogenarians and non-octogenarians, with age-related differences in mortality observed only in Penn class A. These differences may relate to rapid interhospital transfer, surgical strategy, or population characteristics, although the lack of procedural and risk-adjustment data limits interpretation. Nevertheless, the absence of age-related mortality differences in Penn classes B, C, and BC suggests that outcomes in malperfusion states are driven primarily by ischaemic severity rather than age.

### Conservative treatment in octogenarians with ATAAD

Advanced age, shock on admission, and neurological deficit are key considerations when evaluating octogenarians for surgery, as these factors were associated with higher mortality in conservatively managed patients.

Daily activity, cognitive function, and frailty should inform surgical decisions, as sarcopenia is linked to lower home discharge,^26^ although these data were unavailable in our registry.

The spline analysis demonstrated a marked increase in mortality risk beyond approximately 80 years of age, supporting the relevance of examining outcomes specifically in patients aged 80 years or older.

### Limitations

This study has several limitations. It was a multicentre retrospective analysis focused on in-hospital outcomes, and long-term results were not available. Although we adjusted for all clinically relevant variables, residual confounding is possible because factors such as frailty, pre-hospital condition, and treatment selection could not be fully captured. As age ≥80 years is a fixed demographic characteristic, unmeasured differences in frailty, prehospital conditions, or treatment selection may remain despite multivariable adjustment. Therefore, the possibility of unmeasured confounding should be considered. Detailed information on surgical and endovascular procedures was unavailable, preventing assessment of their influence on outcomes. Efforts are underway to link the registry with the Japan Cardiovascular Surgery Database to incorporate procedural data. Additionally, variables such as frailty and cognition were not collected, which may have influenced decisions.

Established risk assessment tools such as the GERAADA score and transmission electron microscopy classification could not be calculated because several required variables were unavailable, limiting comparisons with other registries.

Finally, although the number of octogenarians undergoing surgery increased over the study period, the small sample sizes in Penn classes B, C, and BC limited our ability to detect subtle differences in physiological severity or temporal changes. In addition, the Penn classification could not be applied to non-surgical octogenarians, and malperfusion patterns in this group remain unknown.

## CONCLUSION

The mortality rate of octogenarian patients with ATAAD was significantly higher than that of non-octogenarians; however, operative mortality among octogenarians remained acceptable despite being higher than that among non-octogenarians. Aortic surgery may be a reasonable option for selected octogenarian patients with ATAAD deemed operable, given the relatively favourable operative mortality in this cohort.

## Supplementary Material

ivag195_Supplementary_Data

## Data Availability

Data for this study were obtained from the Tokyo CCU Network Scientific Committee. Access to the dataset is available from the corresponding author with committee approval.

## ABBREVIATIONS

AAD: acute aortic dissection
AAS: acute aortic syndrome
ATAAD: acute Stanford type A aortic dissection
CCU: cardiovascular care unit
CPA: cardiopulmonary arrest
CT: computed tomography
ECMO: extracorporeal membrane oxygenation
GERAADA: German Registry for Acute Aortic Dissection Type A
IHCPA: in-hospital cardiopulmonary arrest
IMH: intramural hematoma
IRAD: International Registry of Acute Aortic Dissection
JCS: Japan Coma Scale
OHCPA: out-of-hospital cardiopulmonary arrest
RCS: restricted cubic splines
ROSC: return of spontaneous circulation
TAAS: Tokyo Acute Aortic Super-Network
