# Supplementary figures and images for: Outcomes of Acute Type A Aortic Dissection in Octogenarians: Tokyo Acute Aortic Super-Network Registry
Source: Interdiscip Cardiovasc Thorac Surg. 2026 Jul 14;41(7):ivag195. doi: 10.1093/icvts/ivag195 (PMC13384056; doi:10.1093/icvts/ivag195)

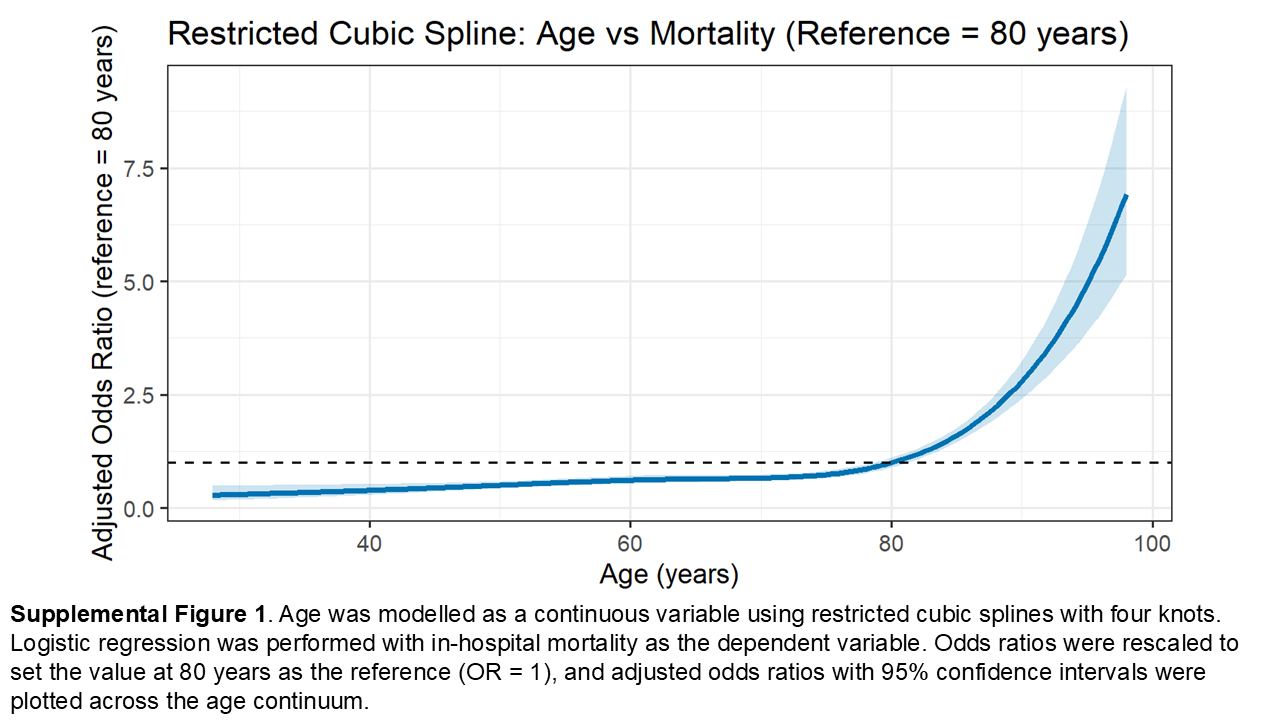

Supplement: ivag195_Supplementary_Data [file ivag195_supplementary_data.zip › SupFig1 with legend.tif]
